# A seed specific dose kernel method for low‐energy brachytherapy dosimetry

**DOI:** 10.1120/jacmp.v4i1.2543

**Published:** 2003-01-01

**Authors:** John J. DeMarco, Timothy D. Solberg, Nzhde Agazaryan

**Affiliations:** ^1^ Department of Radiation Oncology UCLA School of Medicine Los Angeles California 90095

**Keywords:** brachytherapy, TG‐43, Monte Carlo, MCNP, prostate

## Abstract

We describe a method for independently verifying the dose distributions from pre‐ and post‐implant brachytherapy source distributions. Monte Carlo calculations have been performed to characterize the three‐dimensional dose distribution in water phantom from a low‐energy brachytherapy source. The calculations are performed in a voxelized, Cartesian coordinate geometry and normalized based upon a separate Monte Carlo calculation for the seed specific air‐kerma strength to produce an absolute dose grid with units of ${\text{cGy hr}}^{-1}U^{-1}$. The seed‐specific, three‐dimensional dose grid is stored as a text file for processing using a separate visual basic program. This program requires the coordinate positions of each seed in the pre‐ or post‐plan and sums the kernel file for a three‐dimensional composite dose distribution. A kernel matrix size of 81×81×81 with a voxel size of $1.0\times 1.0\times 1.0{\;\text{mm}}^{3}$ was chosen as a compromise between calculation time, kernel size, and truncation of the stored dose distribution as a function of radial distance from the midpoint of the seed. Good agreement is achieved for a representative pre‐ and post‐plan comparison versus a commercial implementation of the TG‐43 brachytherapy dosimetry protocol. © *2003 American College of Medical Physics.*

PACS number(s): 87.53.‐j, 87.52.‐g

## INTRODUCTION

The AAPM Task Group 43 dosimetry protocol (TG‐43) has become an accepted standard of practice for characterizing the dosimetric properties of ${}^{125}I$, ${}^{103}\text{Pd}$, and ${}^{192}\text{Ir}$ brachytherapy sources.^1^ Per TG‐43, the dose rate *D*(*r, θ*), at point (*r, θ*) can be written as

(1) $$\dot{D}(r,\theta )=S_{K}\Lambda \frac{G(r,\theta )}{G(r_{o},\theta_{o})}g(r)F(r,\theta ),$$

where $S_{k}$ is the air‐kerma strength of the source, Λ is the dose rate constant, *G*(*r, θ*) is the geometry factor, *g*(*r*) is the radial dose function, and *F*(*r, θ*) is the anisotropy function. The point $\left(r_{o}=1.0\;\text{cm},\theta_{o}=\pi /2\right)$ is defined at a radial distance of 1.0 cm on the transverse bisector of the source. A commercial treatment planning system will typically implement the TG‐43 protocol using one of three methods: point source approximation, line source approximation, or a two‐dimensional along‐away table. With respect to quality assurance of a commercial brachytherapy treatment planning algorithm, the recommendations of TG‐40 and TG‐64 emphasize the need for an independent calculation prior to implantation that will verify the TG‐43 calculation for at least one location based upon the implant seed distribution.^2^,^3^ This paper provides an alternative calculation method for quality assurance purposes using a Monte Carlo based, brachytherapy seed‐specific dose kernel. The dose kernel is normalized using the simulated air‐kerma strength for each seed and stored as part of a visual basic program that sums the total dose distribution from a collection of seeds. We present an overview of the method, including details of the kernel simulation and implementation techniques used to obtain results for qualitative comparison against those from a conventional brachytherapy treatment planning system.

## METHODS

### A. The Monte Carlo code

The Monte Carlo *N*‐particle (MCNP version 4C) code was used to calculate the three‐dimensional dose distribution from a commercial ${}^{125}I$ brachytherapy seed. The MCNP4C Monte Carlo code is a general‐purpose code capable of simulating coupled neutron‐photon‐electron problems using a three‐dimensional heterogeneous geometry system.^4^ A detailed overview of MCNP4C low‐energy photon physics interactions and cross‐section modifications has been described in a previous study.^5^ The standard MCNP4C cross‐section library (DLC‐200) was updated to include the photoelectric cross‐sections for Z=1–14,19,20,22,46,47 using the more recent DLC‐146 tabulation.^6^ All simulations were performed in the coupled photon‐electron transport mode (mode p e). A kerma tally was used to calculate the collision kerma rate in water and the air‐kerma rate. The MCNP ^*^f4 tally will score the photon kerma by scoring a track‐length estimate of the energy fluence and multiplying this value by an energy dependent mass energy‐absorption coefficient. This tally will yield the absorbed dose assuming local energy deposition of secondary electrons. The mass‐energy absorption coefficients for this study were taken from the calculations of Hubbell and Seltzer.^7^,^8^

### B. Source model

The Syncor Pharmaseed brachytherapy source was chosen as a representative seed design for this study. The Pharmaseed ${}^{125}I$ seed consists of a cylindrical palladium core, 0.325 cm long ×0.05 cm in diameter, onto which a 0.5 *μ*m layer of ${}^{125}I$ has been uniformly adsorbed. The core is sealed within a cylindrical titanium housing 0.45 cm in length ×0.08 cm in diameter. The cylindrical portion of the titanium housing is 0.006 cm thick, with 0.05 cm thick titanium welds at each end. The ${}^{125}I$ decay spectra was taken from the Table of Isotopes. Popescu *et al.* previously measured and calculated the TG‐43 dosimetric parameters for this source,^9^ while DeMarco *et al.* independently verified the TG‐43 parameters using the MCNP4C Monte Carlo code.^10^

### C. Seed specific absorbed dose rate kernel

The three‐dimensional dose distribution in water was calculated based upon a cylindrical phantom geometry of diameter 32 cm and length 32 cm. The cylindrical phantom was discretized into $1\times 1\times 1{\;\text{mm}}^{3}$ voxel elements. The water collision kerma rate was tallied in a cube of 8*$8\times 8{\;\text{cm}}^{3}$, producing a dose matrix of 81×81×81 volume elements. The air‐kerma strength was scored in a separate simulation based upon a cylindrical geometry of 0.2 cm thick×0.2 cm deep at a radial distance of 50 cm from the center of the source. The intervening medium between the source and air‐filled scoring annulus consisted of a vacuum. For the absorbed dose rate and the air‐kerma rate simulations $40\times 10^{6}$ primary particles were followed resulting in statistical uncertainty of less than 0.5% at a distance of 1.0 cm in water and 50.0 cm in air, respectively. The statistical uncertainty increases to approximately 5% at a distance of 4 cm in water. The low‐energy photon and electron cutoff energy was set to 5.0 and 1.0 keV for the water and air‐kerma simulation, respectively. Each voxel in the three‐dimensional tally cube was normalized using the calculated air‐kerma strength to produce a three‐dimensional normalized dose distribution with units of ${\text{cGy hr}}^{-1}U^{-1}$. At 1.0 cm from the transverse bisector of the Pharmaseed source the normalized distribution predicts a value of $0.950\pm 0.005{\;\text{cGy hr}}^{-1}{\;U}^{-1}$. This data point is equivalent to the dose rate constant and is in good agreement with the value of $0.95\pm 0.03{\;\text{cGy hr}}^{-1}{\;U}^{-1}$ and $0.955\pm 0.005{\;\text{cGy hr}}^{-1}{\;U}^{-1}$ calculated by Popescu *et al.* using the MCPT code^9^ and DeMarco *et al.* using the MCNP4C Monte Carlo code.^10^ A Visual Basic program was written to calculate realistic pre‐ and post‐implant brachytherapy dose distributions based upon the normalized dose kernel. The program reads a user‐supplied text file containing the *x*, *y*, and *z* coordinates for each seed in the plan and creates a cumulative dose distribution by summing the three‐dimensional kernel distribution over all seed positions. In the kernel reference frame, the (*x,y,z*) seed coordinate is assumed to lie at the center of the single seed kernel matrix. The transverse axis of the kernel matrix corresponds to the *y* axis in the treatment planning reference frame and assumes that each kernel is perfectly aligned along the superior‐inferior patient direction. This is of course an idealized approximation particularly for post‐implant distributions, since the actual seed orientation will vary based upon the experience of the radiation oncologist or urologist. Based upon user preference the program will output any arbitrary two‐dimensional cut through the cumulative three‐dimensional distribution and compare the results with the conventional brachytherapy treatment planning algorithm. This particular algorithm implements the TG‐43 protocol using a one‐dimensional anisotropy function and assumes a point‐source geometry function.

## RESULTS AND DISCUSSION

**Figure 1:**
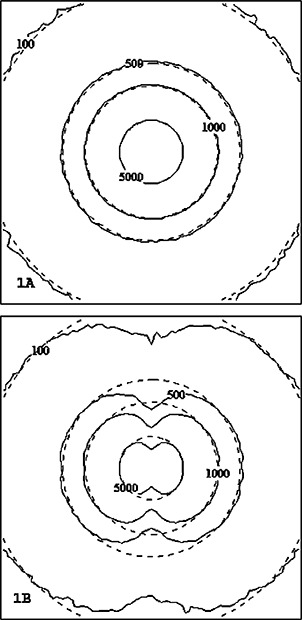
Single seed, absolute dose comparisons of the Monte Carlo dose kernel (solid line) vs TG‐43 point source (dashed line). The absolute isodose lines correspond to the time integrated dose (cGy) based upon an air‐kerma strength of 1.0 U per seed.

**Figure 2:**
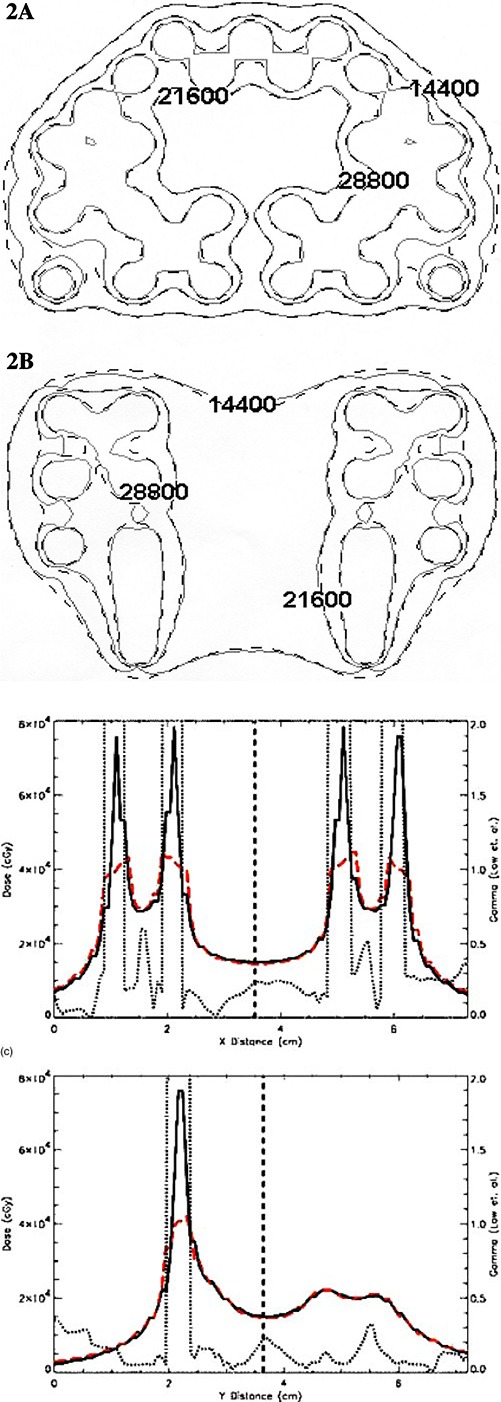
(Color) Comparison of the 129 seed preimplant seed distribution: Monte Carlo dose kernel (solid line) vs TG‐43 point source (dashed line). (a) Axial distribution through the base cut of the prostate. (b) Coronal distribution through the mid‐gland of the prostate. (c) Comparison of the 129 seed pre‐implant seed distribution; Monte Carlo dose kernel (solid line) versus TG‐43 point source (dashed line). Dose profile comparisons based upon arbitrary horizontal and vertical cuts through the axial distribution (a). The dotted line corresponds to the γ calculation of Low *et al.* ^11^ The absolute isodose lines correspond to the time integrated dose (cGy) based upon an air‐kerma strength of 0.43 U per seed.

**Figure 3:**
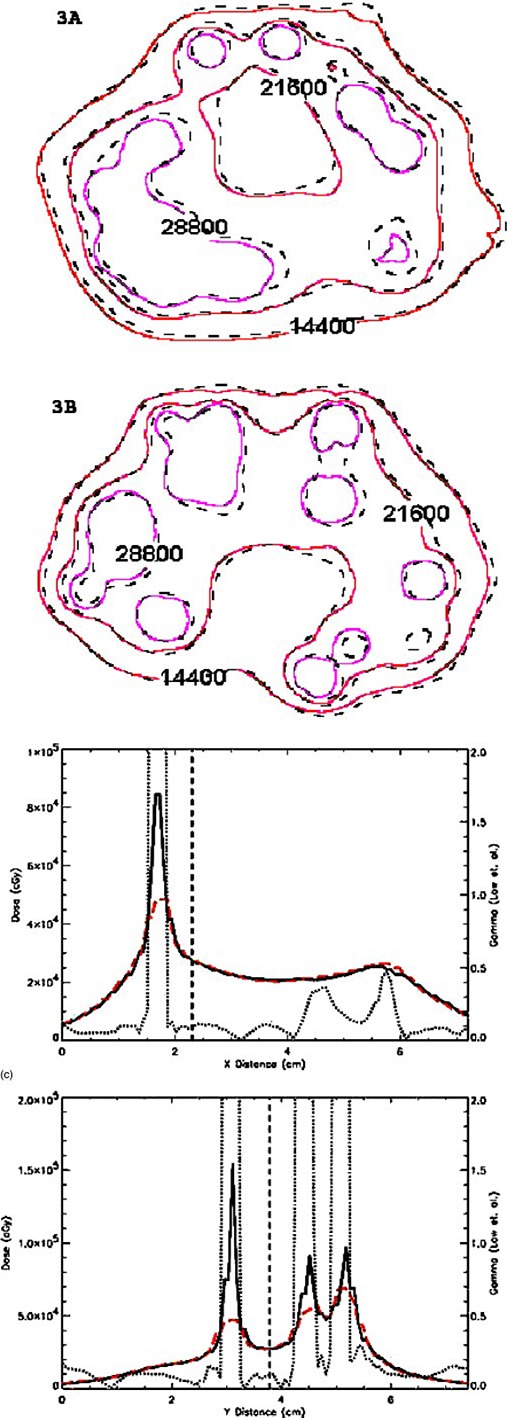
(Color) Comparison of the 120 seed post‐implant seed distribution; Monte Carlo dose kernel (solid line) vs TG‐43 point source (dashed line). (a) Axial distribution through the base cut of the prostate. (b) Coronal distribution through the mid‐gland of the prostate. (c) Comparison of the 120 seed post‐implant seed distribution; Monte Carlo dose kernel (solid line) versus TG‐43 point source (dashed line). Dose profile comparisons based upon arbitrary horizontal and vertical cuts through the axial distribution [The dotted line corresponds to the γ calculation of Low *et al.* ^11^ The absolute isodose lines correspond to the time integrated dose (cGy) based upon an air‐kerma strength of 0.43 U per seed.

**Figure 4:**
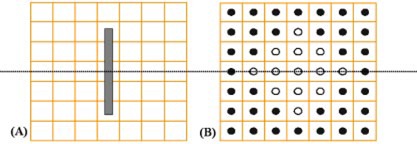
(Color) Dose matrix overview of the TG‐43 implementation as presented for this study vs the Monte Carlo dose kernel. The Monte Carlo dose kernel (a) is based upon a $1\times 1\times 1{\;\text{mm}}^{3}$ tally voxel. A generic cylindrical brachytherapy source with the same length as the BT‐125‐1 source (4.5 mm) is illustrated for scale. The conventional treatment planning calculation matrix (b) is based upon a TG‐43 point source centered within a discrete grid of points with separation of 1 mm. The 12 open circles are assigned the same dose value based upon the value calculated at a radial distance of 2 mm from the point source.

(Figures 1a)and (1b) (axial and coronal cut, respectively) illustrate the comparison dose distribution from one Pharmaseed brachytherapy seed for the MCNP calculation versus the commercial TG‐43 implementation. The mid‐axial distribution produces good agreement between the two methods versus the mid‐coronal cut of the seed. The observed differences at the ends of the seed in the coronal cut are due to anisotropic effects from cladding end welds. This difference is to be expected since the conventional brachytherapy treatment planning algorithm assumes a one‐dimensional anisotropy function for the seed. Figures 2 and 3 illustrate the comparison for a 129 and 120 seed pre‐ and post‐implant source distribution respectively. Each figure compares the calculation methods for an axial (a) and coronal distribution (b). Good qualitative agreement is achieved with the dose kernel summation versus the conventional TG‐43 implementation. (Figures 2c)and (3c) provide a quantitative evaluation based upon one‐dimensional dose profiles through the two‐dimensional axial distributions. The upper and lower parts of each figure correspond to the horizontal and vertical profiles, respectively. Good agreement is achieved with the Monte Carlo kernel method (dashed line) versus the TG‐43 calculation (solid line), except in the vicinity of a seed. The dotted line represents the γ index originally proposed by Low *et al.* for comparing measured versus calculated dose distributions.^11^ The γ index represents a scaled, two‐dimensional distance between a measurement and calculation point determined in combined dose and physical distance space. The γ normalization values used for this study are based upon a maximum percent difference of 3% and a maximum distance‐to‐agreement of 3 mm. The quantitative results illustrated in (Figs. 2c)and (3c) indicate that the conventional treatment planning calculation and the Monte Carlo based kernel summation agree to within 3% or 3 mm for a γ‐index <1.0. For points close to the seed the TG‐43 calculation can underestimate the absolute dose rate by a factor of 2 relative to the Monte Carlo. The particular treatment planning implementation of the TG‐43 point source model limits the user to radial dose points with a minimum distance of 2 mm from the source, while the dose calculation grid can be any arbitrary size. For the TG‐43 point source implemented in the conventional treatment planning system, and a calculation grid spacing of 1 mm, the twelve data points surrounding the point source will be assigned an identical value based upon the calculation point at 2 mm [see (Fig. 4b)]. The Monte Carlo calculated seed kernel is based upon a volume tally, and therefore all voxels receive a dose based upon the average photon track length through the voxel. This also includes the five voxels with a tally volume equal to 1 mm^3^ minus the source segment centered within the voxel [(Fig. 4a)].

## CONCLUSION

In this work we have demonstrated a simple summation algorithm for an independent check of pre‐ or post‐implant brachytherapy seed distributions. A seed specific dose kernel was generated using the MCNP Monte Carlo code and normalized based upon a separate Monte Carlo calculation for the seed specific air‐kerma strength to produce an absolute dose grid with units of ${\text{cGy hr}}^{-1}U^{-1}$. The seed specific kernel distribution is calculated once for each brachytherapy seed design and can be easily distributed via removable media or stored in a central location for download over the internet. This program requires the coordinate positions of each seed in the pre‐ or post‐plan and sums the kernel file for a three‐dimensional composite dose distribution. Good agreement is achieved for a representative pre‐ and post‐plan comparison versus a commercial implementation of the TG‐43 brachytherapy dosimetry protocol based upon a qualitative comparison of isodose lines and the quantitative γ‐index. The greatest discrepancy occurs at calculation points very close to a seed position. The clinical TG‐43 implementation used for this study is based upon a point source and limits the user to radial dose points greater than 2 mm from the seed. This limitation causes the TG‐43 implementation to underestimate the absolute dose rate by up to a factor of 2 relative to the Monte Carlo based summation algorithm for calculation points corresponding to the seed coordinates. The method described could also be applied to other brachytherapy source designs such as ${}^{192}\text{Ir}$ with appropriate modifications to the resolution of the dose matrix and the size of the scoring voxel.

The recommendations of Task Group 64 (Permanent prostate seed implant brachytherapy) are unambiguous regarding independent verification of the conventional treatment planning system for permanent prostate brachytherapy. The task group recommends “*The medical physicist shall verify that the treatment planning system performs the correct dose summation at one or more locations in a simple configuration of multiple seeds*,” and “*Prior to implantation, the dosimetric plan should be checked using an independent procedure or by a second member of the physics staff*….”^2^ While the two‐dimensional isodose comparisons illustrated in this study are not practical for routine quality assurance, the method described could easily calculate the dose at select points within a pre‐ or post‐implant seed distribution. Implementing this method would require easy access to the seed specific dose kernels and the summation program; details currently under consideration as part of a web‐based distribution.

## References

[1] R. Nath , L. L. Anderson , G. Luxton , K. A. Weaver , J. F. Williamson , and A. S. Meigooni , “Dosimetry of interstitial brachytherapy sources: Recommendations of the AAPM Radiation Therapy Committee Task Group No. 43,” Med. Phys. 22, 209–234 (1995). 7565352 10.1118/1.597458

[2] Y. Yu , L. L. Anderson , Z. Li , D. E. Mellenberg , R. Nath , M. C. Schell , F. M. Waterman , A. Wu , and J. C. Blasko , “Permanent prostate seed implant brachytherapy: Report of the American Association of Physicists in Medicine Task Group No. 64,” Med. Phys. 26, 2054–2076 (1999). 10535622 10.1118/1.598721

[3] G. J. Kutcher et al., “Comprehensive QA for radiation oncology: Report of AAPM Radiation Therapy Committee Task Group 40,” Med. Phys. 21, 581–618 (1994). 8058027 10.1118/1.597316

[4] J. F. Briesmeister , “MCNP—A general Monte Carlo N‐Particle transport code, version 4C,” Los Alamos National Laboratory Report No. LA‐12625 (2000).

[5] J. J. DeMarco , R. E. Wallace , and, K. Boedeker , “An analysis of MCNP cross‐sections and tally methods for low‐energy photon emitters,” Phys. Med. Biol. 47, 1321–1332 (2002). 12030558 10.1088/0031-9155/47/8/307

[6] D. K. Trubey , “HUGO IV Photon Interaction Data in ENDF/B‐VI Format,” Radiation Safety Information Computational Center, DLC‐146 (1989).

[7] S. M. Seltzer , “Calculation of Photon Mass Energy‐Transfer and Mass Energy‐Absorption Coefficients,” Radiat. Res. 136, 147–170 (1993). 8248472

[8] J. H. Hubbell and S. M. Seltzer , “Tables of x‐ray mass attenuation coefficients and mass energy‐absorption coefficients (version 1.03), National Institute of Standards and Technology, Gaitherburg, MD; available at http://physics.nist.gov/xaamdi.

[9] C. C. Popescu , J. Wise , K. Sowards , A. S. Meigooni , and G. S. Ibbott , “Dosimetric characteristics of the Pharma Seed™ model BT‐125‐I source,” Med. Phys. 27, 2174–2181 (2000). 11011748 10.1118/1.1289897

[10] J. J. DeMarco , T. D. Solberg , and G. Hugo , “Dosimetric parameters for three low‐energy brachytherapy sources using the MCNP Monte Carlo code,” Med. Phys. 29, 662–668 (2002). 12033560 10.1118/1.1469627

[11] D. A. Low , W. B. Harms , S. Mutic , and J. A. Purdy , “A technique for the quantitative evaluation of dose distributions,” Med. Phys. 25, 656–661 (1998). 9608475 10.1118/1.598248

